# COMMUNITY RESOURCE AWARENESS AND REFERRAL PRACTICES OF HEALTH CARE PROVIDERS TO SUPPORT OLDER ADULTS

**DOI:** 10.1093/geroni/igae098.2869

**Published:** 2024-12-31

**Authors:** Catherine Rudolph, Sarah Francis, Alexandra Bauman

**Affiliations:** Nutrition and Aging Resource Center, Iowa State University, Ames, Iowa, United States; Iowa State University, Ames, Iowa, United States; Iowa Department of Health and Human Services, Des Moines, Iowa, United States

## Abstract

There is mounting evidence that social determinants of health (SDOH), such as housing, income, and food insecurity, have a significant impact on health outcomes. Support for SDOH is vital to ensuring optimal well-being among older adults and preventing unnecessary strain on healthcare resources. Successful partnerships between healthcare and community-based services can help to identify and address the SDOH affecting aging adults’ health. This study examined SDOH screening and intervention practices among healthcare providers, and their awareness and partnerships with community-based food and nutrition services. A nationwide online survey was completed by 160 U.S. healthcare providers who provide care to adults over 60. Descriptive statistics were conducted to assess screening and referral practices. Associations between these practices and provider characteristics are being assessed via Chi-square analyses. SDOH screening was highest for healthcare access, mental health, and food insecurity and lowest for social isolation and transportation. Respondents were most aware of and referred older patients to food pantries, the Supplemental Nutrition Assistance Program, and nutrition education. They were least aware of the Senior Farmers’ Market Nutrition Program, Congregate Meal Program, and nutrition counseling. A majority shared that their healthcare practice has a protocol for community-based referrals. Paper handouts and verbal information were primary referral methods. Common referral barriers were staffing shortages and lack of program access. Over one-half expressed high/very high interest in learning more about community-based food and nutrition services. These findings can inform strategies and opportunities for partnership between healthcare and community-based services to address the needs of aging adults.

